# Validation of self-applied unattended polysomnography using Somte V2 PSG (Somte) for diagnosis of obstructive sleep apnoea (OSA) in pregnant women in early to mid-gestation

**DOI:** 10.1007/s11325-024-03025-0

**Published:** 2024-04-25

**Authors:** Frances Clements, Angela Makris, Yewon Chung, Jonathon Poh, Nathaniel S. Marshall, Kerri Melehan, Renuka Shanmugalingam, Annemarie Hennessy, Hima Vedam

**Affiliations:** 1 Department of Respiratory and Sleep Medicine, Liverpool Hospital, Locked Bag 7103, Liverpool BC, NSW 1871 Australia; 2 School of Medicine, Western Sydney University, Campbelltown, New South Wales Australia; 3 Women’s Health Initiative Translational Unit, Ingham Institute for Medical Research, South Western Sydney Local Health District, Liverpool, New South Wales Australia; 4 School of Clinical Medicine, South Western Sydney Clinical Campuses, Discipline of Medicine, UNSW Sydney, Sydney, New South Wales Australia; 5 Woolcock Institute of Medical Research, Centre for Sleep and Chronobiology, Macquarie University, Sydney, New South Wales Australia; 6 Department of Health Sciences, Macquarie University, Sydney, Australia; 7 Faculty of Medicine and Health, University of Sydney, Sydney, Australia; 8 Department of Respiratory and Sleep Medicine, Royal Prince Alfred Hospital, Camperdown, New South Wales Australia

**Keywords:** Polysomnography, PSG, Pregnancy, Obstructive sleep apnoea, OSA

## Abstract

**Purpose:**

Polysomnography (PSG) may be completed in the home environment (unattended), and when self-applied, allow the collection of data with minimal healthcare worker intervention. Self-applied, unattended PSG in the home environment using Somte PSG V2 (Somte) has not been validated in pregnant women in early to mid-gestation. We undertook a study to evaluate the accuracy of Somte compared to attended PSG. The agreement between apnoea hypopnea index (AHI) and respiratory disturbance index (RDI) scores in Somte and PSG in early to mid-gestation were assessed.

**Methods:**

Pregnant women (≤ 24 weeks gestation) were scheduled for PSG and Somte within a 7-day window, in any order. Somte were self-applied and completed in the home. Somte were scored blinded to PSG result. AHI was the primary outcome of interest, though an AHI ≥ 5 or RDI ≥ 5 on PSG was considered diagnostic of Obstructive Sleep Apnoea (OSA). AHI, RDI, sensitivity, specificity, positive predictive value (PPV), negative predictive value (NPV) was calculated and receiver operating characteristic (ROC) curves were produced. Bland–Altman plots were used to determine agreement. Technical issues occurring during tests were explored.

**Results:**

Twenty-four participants successfully completed both tests between March 2021 and January 2023. PSG were completed at around 14.1 weeks’ gestation (IQR 13.4, 15.7). The time interval between Somte and PSG was a median of 4 days (IQR 2, 7 (range 1–12)). Five (20.8%) women had OSA on PSG at AHI ≥ 5 and 10 (41.6%) women had OSA on PSG at RDI ≥ 5. Somte and PSG did not differ in the measurement of AHI ((1.8, 1.6, *p* = 0.09) or RDI (3.3, 3.5), *p* = 0.73).

At AHI ≥ 5, diagnostic test accuracy (area under the ROC curve) of Somte was 0.94, sensitivity 80.0%, specificity 94.7%, PPV and NPV were 80.0% and 94.7% respectively. At RDI ≥ 5, diagnostic test accuracy (area under the ROC curve) was 0.95, sensitivity 60.0%, specificity 93.0% and PPV and NPV were 85.7% and 76.4% respectively. The confidence limits of Bland–Altman plots were 6.37 to − 8.89 at cut off AHI ≥ 5 and 8.89 to − 10.43 at cut off RDI ≥ 5. Somte failed to start in four tests. Technical issues were reported in both Somte (*n* = 13, 54.2%) and PSG (*n* = 6, 25.0%).

**Conclusion:**

Self-applied, unattended Somte may provide an acceptable substitute to attended PSG in the identification of OSA in pregnant women in early to mid-gestation in this small sample but may fail to detect cases of OSA, particularly when using RDI as the diagnostic marker.

**Supplementary Information:**

The online version contains supplementary material available at 10.1007/s11325-024-03025-0.

## Introduction

### Background rationale

The prevalence of obstructive sleep apnoea (OSA) in the first trimester of pregnancy ranges from 3.6% to 10.5% of pregnancies [1, 2] and increases in obese individuals [2]. A recent consensus statement has recommended screening for OSA in early gestation in pregnant women with body mass index (BMI) ≥ 30, or a history of diabetes and/or hypertensive disorder(s) of pregnancy (HDP) in the current or previous pregnancy [3].

Options available for screening or detecting OSA include simple screening devices (level IV), limited channel devices (level III), unattended PSG (level II) or gold-standard attended PSG (level I). The Apnealink Air (Apnealink Air, Resmed, Sydney, Australia), a level III test, has been validated in early to mid (≤ 24 weeks) [4], and later gestation (≥ 28 weeks) [5]. However, the Apnealink device does not record electroencephalogram (EEG) data, from which arousals can be determined. Cortical arousals are required to identify respiratory events such as apnoeas and hypopnoeas and Respiratory Effort-Related Arousal when there is not an associated desaturation event. This is relevant for pregnant women in whom the diagnosis of OSA may rely on arousals rather than desaturations given their young age, relative lack cardiorespiratory comorbidities, and pre-test probability of having mild rather than severe disease.

Level I and II tests include collection of EEG, but are expensive tests to conduct and require experienced technicians [6]. Level II devices may be completed in the sleep laboratory or home setting though will typically use technicians for the set-up process, regardless of the intended location of testing. It is possible, however, for level II studies to be self-applied by the patient in the home environment, and this may improve accessibility and reduce wait times, allowing earlier testing of pregnant women at risk of OSA related pregnancy complications. Access to sleep testing during early gestation may be important, as the early gestational period represents a potential window for intervention among gravid women with confirmed OSA who are at risk of pregnancy complications. Placentation completes at approximately 16 weeks’ gestation, and preventative therapies such as aspirin, when commenced by 16 weeks’ gestation, has demonstrated a reduction in preeclampsia risk [7]. Therefore, level II PSG, self-applied in the home environment, provides an opportunity for timely access to testing in pregnancy. This early diagnosis may be crucial in allowing timely intervention to allow for effective prevention of pregnancy related complications.

The aim of this study was to assess the agreement in the apnoea hypopnea index (AHI) and respiratory disturbance index (RDI) of level II unattended, self-applied Somte PSG V2 (Somte) and level I attended PSG (PSG) in early to mid-gestation. We also assessed the feasibility of self-application of Somte in the home environment.

## Methods and analysis

Our report follows Standards for Reporting Diagnostic Accuracy (STARD) guidelines for reporting diagnostic studies [8] (Online supplement 1).

### Study design and setting

During screening for eligibility in a pilot randomised controlled trial (RCT) (ANZCTR number ANZCTRN12619001530112) which commenced in September 2019, pregnant women were invited to undertake level III Apnealink Air in the home environment, and level I attended PSG in a hospital sleep investigation unit (SIU) by the 24th week of gestation. SARS-CoV-2 pandemic public health orders in South-Western Sydney during 2020 resulted in the closure of the SIU and temporary suspension of the study and we were unable to complete attended PSG during this time. During the study recruitment pause we developed an amended protocol to include validation of an additional sleep diagnostic test (Somte). Somte were to be completed in the home environment using self-application method to reduce staff exposure to patients, which was the public mandate at the time. Data were to be compared to gold-standard attended PSG. The amendment was approved in January 2021, implemented in March 2021 and the first Somte study collected 28th March 2021. A further SARS-CoV-2 study suspension and SIU closure restricted access to attended PSG between July and December 2021. Sample size target for this study was not predetermined. The intention was to collect as many paired studies as possible during the study collection period. Recruitment for this study concluded in January 2023.

Participants were recruited from antenatal clinics at Campbelltown and Liverpool hospitals in South Western Sydney, NSW, Australia. Somte tests were completed in the participant home. Attended PSG were completed in the SIU at Liverpool Hospital. Participants diagnosed with OSA on PSG were offered participation in the RCT study if eligible. Participants not eligible for RCT participation but who were deemed by the sleep physician to require treatment were offered CPAP through a clinical pathway.

### Participants

At or prior to scheduled hospital obstetric bookings, participants were approached in person or by phone in a convenience series by study staff who completed screening eligibility using REDCap [9]. Eligible participants were provided a participant information sheet by email or hard copy and provided informed written consent if eligible to participate. Baseline data were collected using REDCap questionnaire, including STOP BANG [10], Epworth Sleepiness Scale (ESS) [11] and pregnancy-related tool questionnaires [12]. Gestation was based on the dating scan performed in the first trimester. Participants undertook both the Somte and PSG according to availability of participant and test devices which were organised by the study coordinator. Both studies were scheduled within a target period of 7 days. Only participants who successfully completed both tests within 14 days were included for analysis.

Inclusion criteria were defined as (1) women aged 18 years of age and above; (2) In early-mid pregnancy (up 24 weeks gestation); (3) at increased risk of metabolic complications defined as ONE OR MORE of: (a) body mass index (BMI) greater than or equal to 35 kg/m^2^ at screening; (b) previous Gestational diabetes mellitus (GDM) [13]; (c) previous personal history of pre-eclampsia (or in mother or sister); (d) underlying renal disease; (e) maternal type 2 diabetes (pre-gestational); (f) symptoms of sleep disordered breathing (SDB) including snoring, witnessed apnoea, mild excessive daytime sleepiness (EDS) (which does not meet the criteria for severe EDS defined by Epworth Sleepiness Scale (ESS) (> 15) or a fall asleep accident or near-miss accident in the previous 12 months) or tiredness. Participants were excluded if they have (1) previous diagnosis of OSA on active treatment; (2) confirmed current GDM or preeclampsia; (3) maternal type 1 diabetes; (4) multifetal gestation, (5) known fetal chromosomal abnormality; (6) inability to provide informed consent; (7) severe EDS based on clinical assessment (e.g. including a fall asleep motor vehicle accident or near miss, transient sleepiness while driving/at lights or needing to pull over due to sleepiness while driving, or transient sleepiness in any other dangerous situation i.e. cooking, carrying baby) or ESS of greater than 15.

### Attended PSG (reference standard)

**Fig. 1:**
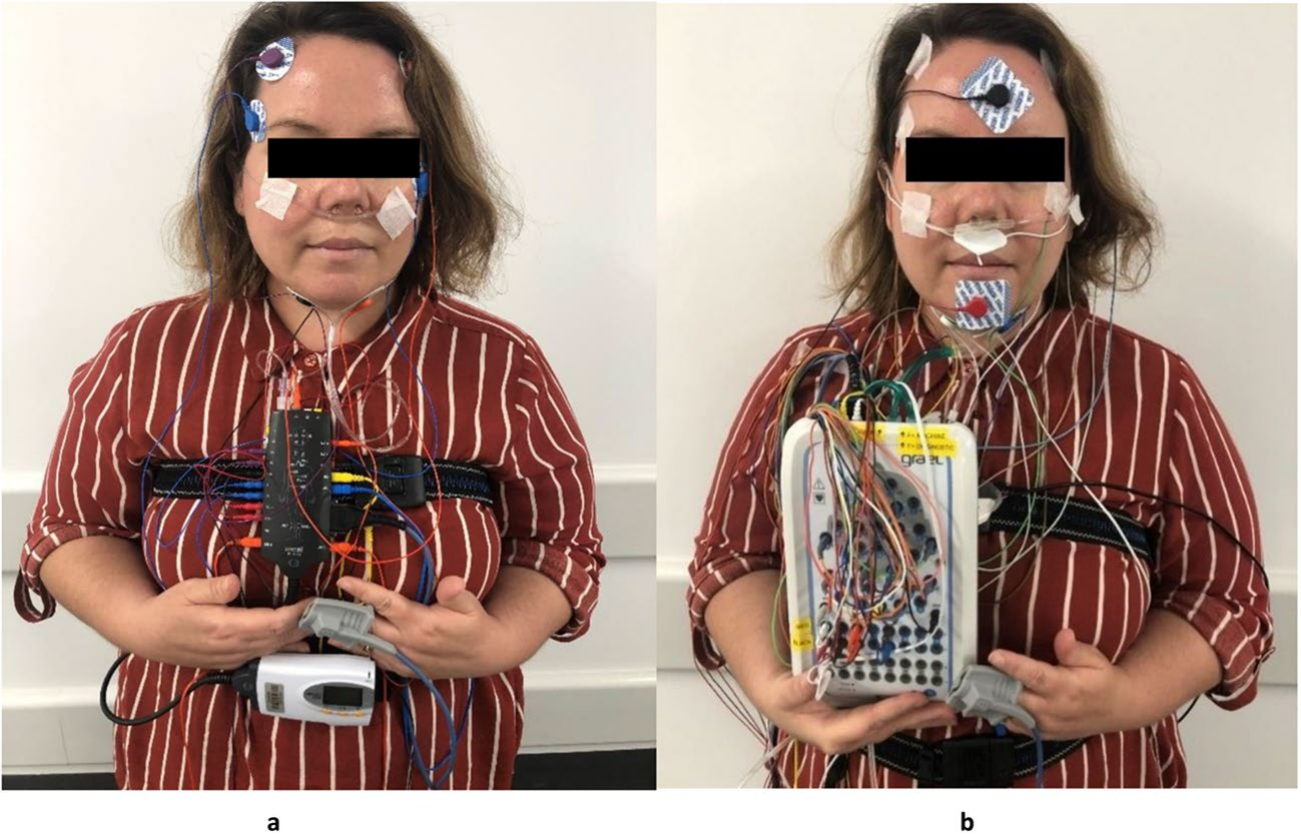
**a**, **b** Devices used in the study. Figure 1a, b demonstrates the first author wearing the Somte and PSG devices. The Somte image does not depict the thermistor, which was used during the test. The Somte was completed in the participant home, following self-application of the test. The PSG was completed in the sleep investigations unit at Liverpool Hospital, following technician application of the test

Participants completed PSG (Grael 4 K PSG: EEG, Grael Acquisition system, Compumedics, Abbotsford, Australia). Participants had routine observations on arrival (BP, pulse, Sp02, height, weight) and if there were any clinically significant abnormalities, an obstetric physician was called. Participants were set up by experienced sleep technicians. PSG data were collected between 21:00–06:00 approximately. PSG data collection followed international 10–20 system and included EEG (C1/C2 C3/C4, O1/O2, F3/F4, M1/M2), EOG (E1/E2), EMG (chin, diaphragm and anterior tibialis (left and right)), snore (microphone), ECG (modified Lead II), airflow (pressure transducer, thermistor), respiratory effort (abdomen and thoracic), oximeter (SpO_2_), sound level (dB meter), digital video (audio and visual) and position sensor (Fig. 1b). SpO_2_ was collected using Compumedics adult silicone soft tip probe- oximeter- 3 m. Time available for sleep (TAS) represents recording time in minutes between lights on and off / off and on, as visualised in study video. Total sleep time (TST) represents sleep time in minutes.

### Somte (index test)

Participants were issued the Somte (Compumedics, Abbotsford, Australia) and manufacturer instructional booklet by the study coordinator at the consent visit if the test was scheduled to be completed on the night of the consent visit, or on the date of the Somte study, if it was scheduled to be completed later. Participants were encouraged to watch a manufacturer produced YouTube instructional video prior to undertaking the test [14]. The study coordinator demonstrated the Somte device operation and correct application technique for the nasal cannula, thermistor, oximeter, and abdominal and thoracic effort bands. Participants self-applied the Somte device in the home environment. Assistance from spouse or partner, during the set-up of Somte, was allowed, but not encouraged.

Somte data collection commenced using either manual or pre-specified start options. Initially, participants manually started the Somte at their preferred sleep time, using the manual start function on the device. However, due to failure to start in four Somte studies, start times were subsequently preprogramed according to the anticipated sleep time, as reported by the participant on the day of device collection. The study coordinator was available for technical phone support during the sleep study set-up and data collection. Participants were instructed to manually stop the test at end of sleep period (sleep offset).

Somte data collection followed a modified American Academy of Sleep Medicine (AASM) set-up protocol. Participants self-applied EEG (F3/F4, M1/M2), electrooculography (EOG (E1/E2)), EMG chin (submentalis, anterior tibialis (left and right leg), electrocardiogram (ECG (modified Lead II)), airflow (pressure transducer), snore, airflow (thermistor), respiratory effort (abdomen and thoracic), oximeter (SpO_2_) and position sensor. F3/4 leads were placed on the forehead for ease of application by the participant (Fig. 1a). SpO_2_ was collected using Compumedics adult silicone soft tip probe- oximeter- 1 m. Time available for sleep (TAS) represents recording time in minutes of the duration of recording between which the scorer deemed the patient to have attempted to sleep, and was determined using a combination of position change, signal quality and movement. Our study did not collect patient reporting of sleep onset and sleep offset time via questionnaire. Total sleep time (TST) represents sleep time in minutes.

### Scoring and reporting of studies

Somte and PSG were manually scored using version 2.6 2020 AASM manual for the scoring of sleep and associated events [15] using Profusion PSG 4 software (Compumedics, Abbotsford, Australia). Event definitions are defined according to AASM guidelines:

- *Obstructive apnoea*: reduction of ≥ 90% of baseline in airflow for 10 s or more with continued or increased effort throughout.
- *Central apnoea*: reduction of ≥ 90% of baseline in airflow for 10 s or more with absent effort throughout.
- *Mixed apnoea*: reduction of ≥ 90% of baseline in airflow for 10 s or more with absent effort initially followed by resumption of effort in the second portion.
- *Hypopnoea*: reduction of ≥ 30% of baseline in airflow for 10 s or more accompanied by either
- an arousal or 3% desaturation.
- *Respiratory Effort-Related Arousal (RERA)*: a sequence of breaths lasting ≥ 10 s characterized by increasing effort or by flattening of the inspiratory portion of the flow leading to an arousal from sleep.
- *AHI*: Apnoeas and hypopnoeas only.
- *Respiratory Disturbance Index (RDI)*: AHI + RERA.

Somte were de-identified prior to data collection. Following data collection, Somte raw data were downloaded from the device by the study coordinator and scored and reported blinded to the PSG result. A single, experienced sleep scientist manually scored all Somte and PSG. Following scoring, one of two sleep physicians who were not involved in the direct care of respective patients, reported studies. The study coordinator was unblinded to identified participant sleep data though was not involved in the scoring or reporting of any Somte or PSG studies.

### Statistical methods

Data were entered into REDCap. Analysis was performed using Statistical Package for the Social Sciences (SPSS), Version 29.0 (IBM Corp., Chicago, Illinois, USA). Data were checked for errors in SPSS by calculating minimum and maximum values for each variable and checking for outliers or anomalies. Descriptive data is presented (based on distribution as assessed by a Shapiro–Wilk test) either as a mean ± standard deviation (SD) or median (interquartile range (IQR)) or count (%). Diagnostic test accuracy measures were undertaken (sensitivity, specificity, positive predictive value (PPV), negative predictive value (NPV)) and by creating receiver operating characteristic (ROC) curves. Time available for sleep (TAS) and total sleep time (TST) of both tests were calculated and reported in minutes. Cohen's kappa coefficient was calculated. Sleep variable data of both tests were calculated and reported in percentage of TST, events per hour, minimum or mean. The calculated AHI and RDI of each method were compared and Bland–Altman plot used to plot limits of agreement. The AHI was the primary outcome of interest though an AHI ≥ 5 or RDI ≥ 5 at PSG was considered diagnostic of OSA for the purpose of eligibility screening for participation in the RCT. Imputation was not used for missing data. Indeterminate results were considered false-positive or false-negative and incorporated into the final analysis.

## Results

**Fig. 2:**
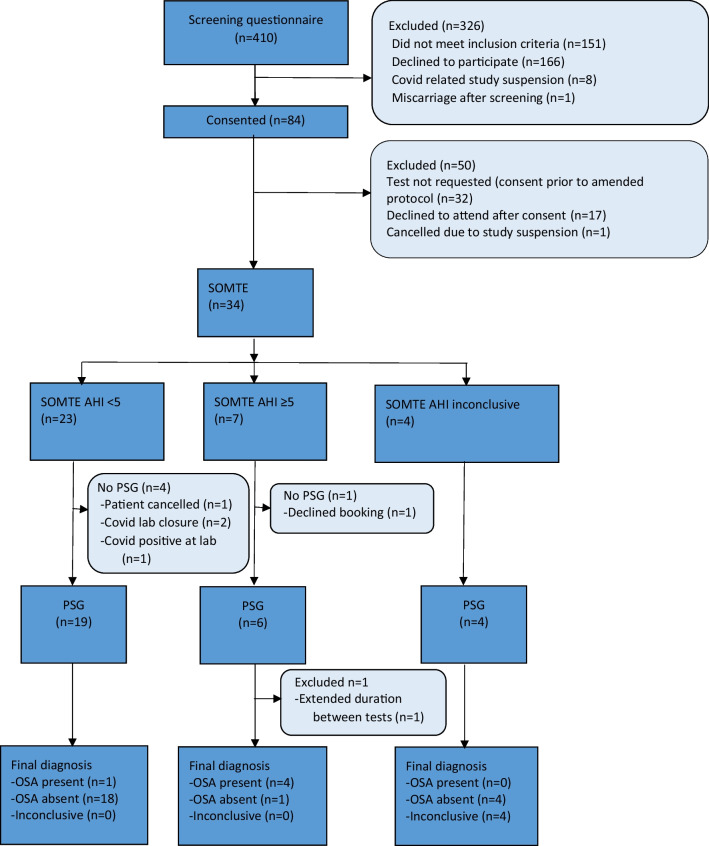
Participant flowchart. Participants were excluded if they did not complete both Somte and PSG tests successfully within a 14-day window. PSG represents polysomnography. AHI represents apnoea hypopnoea index

A total of 410 participants were screened for eligibility and eighty-four participants consented for participation. Thirty-two consented participants were ineligible to complete Somte as they were recruited prior to Somte addition to the study protocol. Of the fifty-two consented participants recruited after addition of Somte to the study protocol, thirty-four participants underwent a Somte, and twenty-nine participants completed PSG. Both Somte and PSG were attempted by twenty-nine participants and of these, one was excluded due to an extended duration of time between tests (17 days) and four excluded due to Somte failure to start. Subsequently, twenty-four participants are included for data analysis. The study flow chart is shown in Fig. 2. Baseline demographics data of study participants (*n* = 24) is shown in Table 1.

**Table 1:** Participant baseline demographics (*n* = 24)

Parameter	Mean ± SD, median (IQR) or count (%)	
Age	33.6 ± 6.2	
BMI	31.5 ± 7.1	
BMI ≥ 30	13 (54.2%)	
Systolic blood pressure (mmHg)	116.8 ± 10.5	
Diastolic blood pressure (mmHg)	71.0 ± 8.1	
Personal history of hypertension	10 (41.7%)	
Gravidity	2 (1.8, 3.6)	
Nulliparous	10 (41.7%)	
STOP-BANG total score	1.9 ± 1.0	
ESS score	6.8 ± 3.5	
Pregnancy-specific screening tool total score	78.2 ± 15.4	
Pregnancy-specific screening tool positive score (score ≥ 75)	16 (66.7%)	
Personal history GDM	4 (16.7%)	
Family history of diabetes (1st degree relative with diabetes or a sister with gestational diabetes)	12 (50.0%)	
History of preeclampsia in self, mother, or sister	5 (20.9%)	
History of kidney disease	2 (8.3%)	
Self-reported snore at least three times a week	11 (45.9%)	
Witnessed apnoeas	4 (16.7%)	
Previous pregnancy with birth weight 4.5 kg or more	2 (8.3%)	
History of polycystic ovarian syndrome	2 (8.3%)	
Current steroid therapy	0 (0.0%)	
Proteinuria (dipstick assessment)	Negative	14 (67%)
	Trace	5 (24%)
	30 mg/dL ( +)	2 (9%)
Self-reported ethnicity	Caucasian	11 (45.8%)
	Other	5 (20.8%)
	Asian	3 (12.5%)
	Indian/Subcontinental	2 (8.3%)
	Polynesian	1 (4.1%)
	Middle Eastern	1 (4.1%)
	Aboriginal/Torres	1 (4.1%)
	Strait Islander	

*BMI* represents body mass index; *GDM* represents gestational diabetes mellitus; *ESS* represents Epworth sleepiness scale

OSA (AHI ≥ 5) was identified in 5 participants (20.8%) on PSG and 5 participants (20.8%) on Somte. RDI ≥ 5 was identified in 10 participants (41.7%) on PSG and 7 participants (29.2%) on Somte. Percentage of REM achieved during sleep did not differ between Somte (21.3%) and PSG (20.2%) (*p* = 0.179). The median (IQR) and range (minimum, maximum) of Somte and PSG (AHI and RDI), and sensitivity, specificity, PPV, NPV and area under ROC curve (AHI ≥ 5 and RDI ≥ 5) is shown in Table 2. Likelihood ratio for AHI ≥ 5 for Somte compared to PSG was 15.20 (positive) (95% CI 2.15 to 107.63) and 0.21 (negative) (95% CI 0.04 to 1.22), Likelihood ratio for RDI ≥ 5 for Somte compared to PSG was 8.40 (positive) (95% CI 1.19 to 59.36) and 0.43 (negative) (95% CI 0.20 to 0.93).

**Table 2:** Results (*n* = 24)

Test	Median (IQR) / range	Sensitivity (95% CI)	Specificity (95% CI)	PPV (95% CI)	NPV (95% CI)	Area under ROC curve (95% CI)
PSG AHI events/hour	1.6 (0.5, 4.6) / 0.0 – 18.1	Reference Test PSG: cut off AHI ≥ 5 events/hour				
SOMTE AHI events/hour	1.8 (0.5, 4.6) * / 0.0 – 26.9	80.0% (95% CI 28.4% – 99.5%)	94.7% (95% CI 74.0% – 99.9%)	80.0% (95% CI 36.1% – 96.6%)	94.7% (95% CI 75.7%—99.1%)	0.94 (95% CI 86.1% - 100%)
PSG RDI events/hour	3.5 (1.0, 9.5) # / 0.1 – 19.5	Reference Test PSG: cut off RDI ≥ 5 events/hour				
SOMTE RDI events/hour	3.3 (0.7, 6.4) / 0.0 – 29.8	60.0% (95% CI 26.2%—87.8%)	93.0% (95% CI 66.1%—99.8%)	85.7% (95% CI 45.9%—97.7%)	76.4% (95% CI 60.0%—87.6%)	0.95 (95% CI 85.2% – 100.0%)

AHI and RDI median, inter quartile results (IQR) / range of Somte and PSG. Sensitivity, specificity, positive predictive values (PPV) negative predictive values (NPV) and area under the receiver operating curve (ROC) AHI ≥ 5 events/hour and RDI ≥ 5 events/hour with 95% confidence intervals (95% CI). PSG represents polysomnography. AHI represents apnoea hypopnea index. RDI represents respiratory disturbance index. * *p*-value 0.09. # *p*-value 0.73

PSG were completed at around 14.1 weeks’ gestation (IQR 13.4, 15.7). The time interval between Somte and PSG was a median of 4 days (IQR 2, 7; range 1–12). The median TAS for Somte and PSG was 461.8 min (IQR 386.6, 506.5) and 526.3 (IQR 499.1, 535.4) (*p* =  < 0.001) respectively. The Median TST for Somte and PSG was 391.3 (IQR 320.4, 429.1) and 438.2 (IQR 345.4, 475.9) (*p* = 0.10), respectively. Sleep study Median (IQR) of AHI and RDI of Somte and PSG, and additional sleep variable data are shown in Table 3.

**Table 3:** Sleep variables for Somte and PSG study data (*n* = 24)

	Somte (Median (IQR)	PSG (Median (IQR)	Significance (*p*-value)
Time available for sleep (minutes)	461.8 (386.6, 506.5)	526.3 (499.1, 535.4)	< 0.001
TST (minutes)	391.3 (320.4, 429.1)	438.2 (345.4, 475.9)	0.10
Percentage of TST in REM sleep %	21.3 (16.6, 23.9)	20.2 (16.1, 21.9)	0.179
Percentage of TST in N1 sleep %	5.4 (2.9, 7.7)	7.7 (4.6, 10.6)	0.14
Percentage of TST in N2 sleep %	49.9 (43.4, 55.1)	56.5 (50.3, 61.5)	< 0.001
Percentage of TST in N3 sleep %	22.0 (16.5, 33.6)	17.6 (9.7, 23.9)	< 0.001
Overall AHI events/hour	1.75 (0.5, 4.6)	1.6 (0.5, 4.4)	0.94
Overall RDI events/hour	3.3 (0.7, 6.4)	3.5 (1.0, 9.5)	0.726
Overall ODI events/hour	2.1 (0.6, 5.0)	0.7 (0.2, 3.6)	< 0.001
Percentage of TST in SUPINE sleep	42.8 (18.2, 52.6)	44.5 (21.9, 62.4)	0.445
SUPINE AHI events/hour	2.2 (0.1, 7.8)	3.4 (0.4, 9.4)	0.626
SUPINE RDI events/hour	2.8 (0.7, 8.9)	6.8 (0.7, 12.2)	0.306
SUPINE ODI events/hour	4.5 (0.7, 6.8)	1.5 (0.7, 4.2)	0.068
NON-SUPINE AHI events/hour	0.7 (0.0, 2.2)	0.7 (0.0, 1.8)	0.070
NON-SUPINE RDI events/hour	1.5 (0.0, 3.4)	1.7 (0.6, 5.8)	0.974
NON-SUPINE ODI events/hour	0.8 (0.3, 3.7)	0.4 (0.0, 2.6)	0.004
Minimum SpO2	90.0 (89.0, 92.0)	92 (89.3, 94.0)	0.004
Mean (average) SpO2	96.0 (95.0, 96.0)	97.0 (96.0, 97.0)	< 0.001
Percentage of TST with SpO2 < 90%	0.0 (0.0, 0.1)	0.0 (0.0, 0.0)	0.011

Results presented as median with interquartile range (IQR) and significance (*p*-value). *PSG* represents attended polysomnography; *TST* represents total sleep time; *AHI* represents apnoea hypopnea index; *RDI* represents respiratory disturbance index; *ODI* represents oxygen desaturation index

**Fig. 3:**
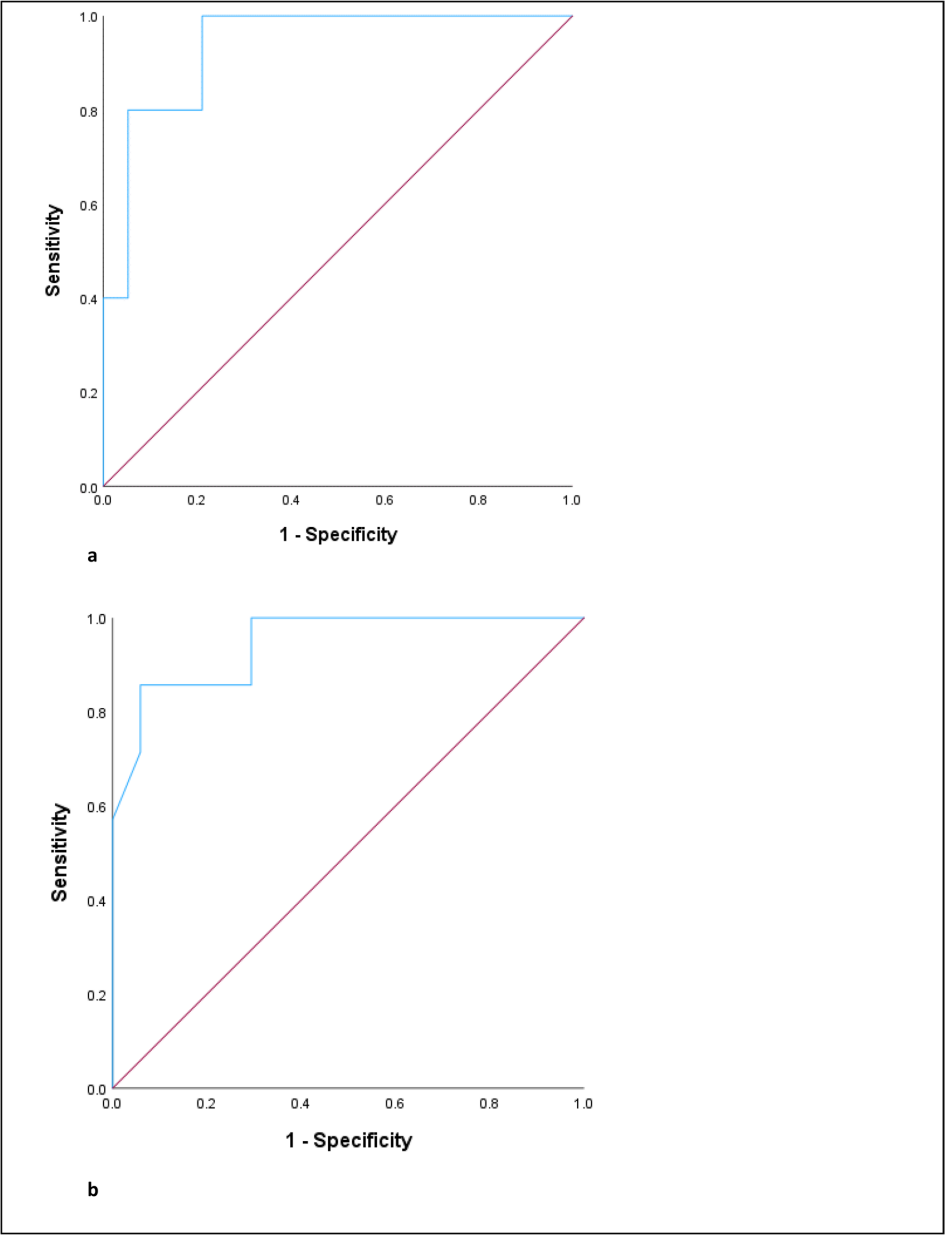
**a**, **b** Receiver operating characteristics (ROC) curves for Somte compared to PSG at a) AHI ≥ 5 and b) RDI ≥ 5. The Area under ROC curve (95% confidence interval) of Somte compared to PSG at AHI ≥ 5 is 0.94 (95% CI 86.1%—100%) and RDI ≥ 5 is 0.95 (95% CI 85.2% – 100.0%)

**Fig. 4:**
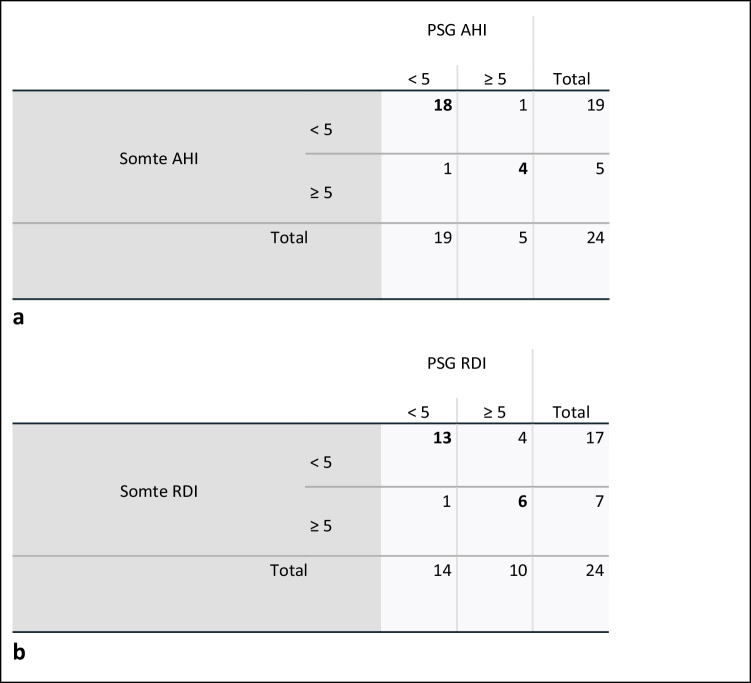
**a**, **b** Contingency tables for Somte and PSG at a) AHI ≥ 5 and b) RDI ≥ 5. PSG represents polysomnography. AHI represents apnoea hypopnea index. RDI represents respiratory disturbance index. The Kappa coefficient for AHI ≥ 5 comparing PSG and Somte is 0.747 ± 0.17, and for RDI ≥ 5 comparing PSG and Somte is 0.552 ± 0.17

**Fig. 5:**
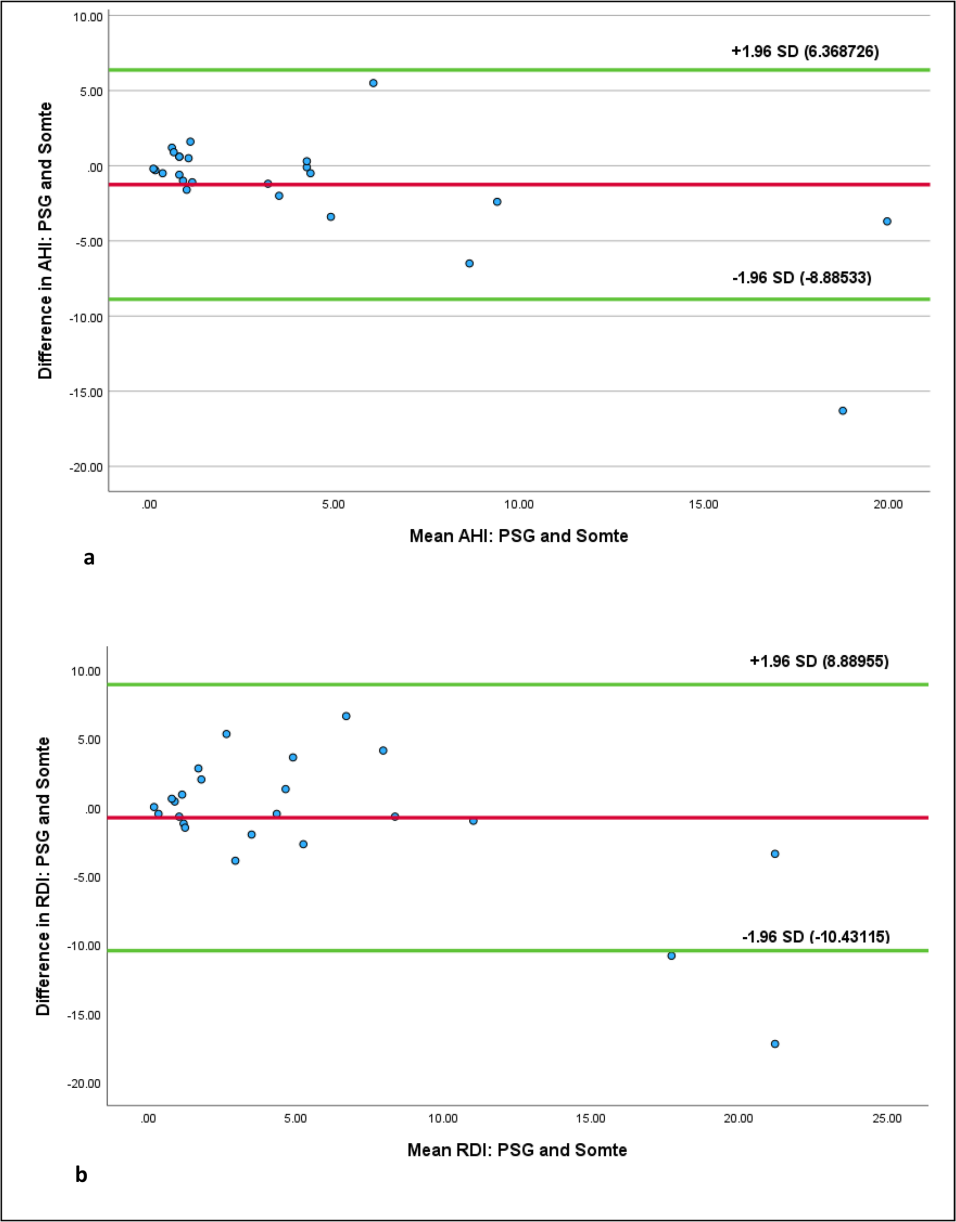
**a**, **b** Bland–Altman plots indicating agreement between a) AHI and b) RDI derived from the SOMTE and the PSG. The solid red line represents the mean difference line, the solid green lines represent the upper lower 95% confidence limits a) 6.37, -8.89 and b) 8.89, -10.43, respectively

ROC curves are shown in Fig. 3a, b. Positive and negative predictive values were calculated using cross tabulation in SPSS and are shown at Fig. 4. The Kappa coefficient for AHI ≥ 5 comparing PSG and Somte is 0.747 ± 0.17, and for RDI ≥ 5 comparing PSG and Somte is 0.552 ± 0.17. Bland–Altman plots for AHI ≥ 5 and RDI ≥ 5 are shown at Fig. 5a, b. No adverse events were reported by participants undertaking Somte and/or PSG.

One participant displayed increased muscle tone on chin EMG during rapid eye movement (REM) on Somte and PSG. Additionally, the participant demonstrated frequent motor activity (subtle head movements and one episode of body jerking) during each REM period, as evidenced on video PSG, and was subsequently diagnosed with REM sleep without atonia (RSWA).

In addition to the four Somte studies that failed to start, technical issues were seen in both Somte (*n* = 13, 54.2%) and PSG (*n* = 6, 25.0%), *p* = 0.41 (Fisher’s exact test). Somte reported signal quality loss in flow (*n* = 3, 12.5%), SpO_2_ (*n* = 3, 12.5%), EEG (*n* = 3, 12.5%), EMG (*n* = 6, 25.0%), and ECG (*n* = 2, 8.3%). PSG studies reported signal quality loss in airflow (*n* = 1,4.2%), SpO_2_ (*n* = 2, 8.3%), EEG (*n* = 2, 8.3%) and EMG (*n* = 3, 12.5%). None of the signal quality losses, in either PSG or Somte, resulted in a non-diagnostic study. However, 2 Somte studies used surrogate imputations for scoring the study, as permitted in the AASM scoring guidelines.

## Discussion

This work demonstrates Somte can be self-applied by pregnant women, and accurately diagnoses OSA in early to mid-pregnancy. At the clinically significant threshold of AHI ≥ 5, compared to gold standard PSG, Somte demonstrated excellent specificity, NPV, and area under the ROC curve, but had a lower sensitivity and PPV. At RDI ≥ 5, Somte demonstrated a lower specificity, NPV and sensitivity compared to PSG using AHI, though reported a higher area under the ROC curve and PPV. Proportional bias was noted on the Bland–Altman plots, with Somte underestimating AHI and RDI scores at higher severity scores. Technical issues occurred in Somte and PSG, although, except for the Somte which failed to start, these did not preclude the usefulness of the test.

Other studies investigating agreement in AHI scores between Somte and PSG have reported differing findings to our study. Ferré et al. reported similar area under the ROC curve to our study at AHI ≥ 5 of 0.90 vs 0.94 though differences in sensitivity (90.5% vs 80.0) specificity (83.5% vs 94.7%), PPV (96.0% vs 80.0%) and NPV (63.5% vs 94.7%) which may reflect the differences in age, gender, and severity of OSA between the two studies [16]. Cunnington et al. did not compare results at cut off AHI ≥ 5 but at cut off AHI ≥ 10, reported area under the ROC curve of 89.4, sensitivity 96.7%, specificity of 40.0%, PPV 91.2% and NPV 65.4%. This represents a higher sensitivity than our study (96.7% vs 80.0%) but lower specificity (40.0% vs 94.7%) and NPV (65.4% vs 76.4%) [17]. Our study reports lower sensitivity than both Ferré and Cunnington, though higher specificity and this may reflect the different patient population in which the tests are conducted. Further, both Ferré and Cunnington conducted simultaneous tests and use different EEG positions than our study (central vs frontal), which may have resulted in different results. A systematic review investigating portable sleep diagnostic tests report overall support of the use of level II devices, with specificity increasing with increased OSA severity [18]. The low specificity of our study findings may reflect the low AHI scores in our study.

The major strength of this study design is the completion of the index and reference tests during early to mid-gestation. This may improve early access to PSG in the early to mid-gestation period as recommended for at risk women [3]. By using a self-application method, we have been able to demonstrate accuracy compared to attended PSG, for pregnant women requiring sleep diagnostic testing.

Further strengths of this study include use of blinding of the reference test result to minimise bias, the use of a single software package for the scoring of Somte and PSG data, the use of a single scorer across both the reference and index tests and inclusion of pregnant women with and without traditional signs or symptoms of OSA. By including pregnant women with personal or family history of GDM and/or preeclampsia, this makes our data relevant for real life clinical applications, as our participants broadly reflect the population recommended for OSA screening in pregnancy [3].

Four Somte tests failed to start following completion of the set-up process by the participant and the cause was not identified. Subsequent discussions with the manufacturer point to the possibility the device was draining the battery whilst in standby mode. Additional technical difficulties that resulted in signal loss or dropout were reported in 54.2% of our Somte studies although these did not result in test failure. Failure of Somte has been reported previously by Light et.al with 14/221 (6%) of studies failing due to poor oximetry trace (Sp0_2_) when conducted in the home, in non-pregnant adults, following set-up by trained technicians [19]. We note the sleep scientist involved in scoring all Somte and PSG in our study has significant experience in scoring sleep studies and we would caution the use of inexperienced scorers to score unattended PSG of pregnant women who have self-applied the device, due to the high rate of technical issues encountered in our participants, despite extensive education prior to testing, and on-call phone support available during the test.

Our study limited Somte EEG to F3/4 only which was repositioned to the forehead. Use of repositioned F3/4 (with M1/2 referencing) was chosen for ease of application and has been investigated in other studies involving non-pregnant adults. Ferré et.al investigated agreement between Somte with F3/4 EEG only, and simultaneous PSG, demonstrating good agreement in sleep efficiency (68%), specificity (84%), and area under the ROC curve (0.86) at AHI cut off ≥ 5, and high sensitivity (91%), though did report a statistically significant difference in AHI scores [16]. Further, Light et.al investigated scoring of sleep vs wake in non-pregnant adults, using F3/4 only EEG, repositioned to the forehead, similarly to our study. Lights’ study scored a single night of full PSG study twice, one with access to all collected EEG signals, and one with access to only F3/4 (other signals hidden) [19]. The study demonstrated high agreement (94%) in sleep vs wake epochs in studies using only F3/4 compared to those with full EEG. Our study did not complete the tests simultaneously and this may have contributed to the differences in reporting of maximum AHI and RDI scores in Somte studies (26.9, 29.8) compared to PSG (18.1, 19.5). Whilst the overall AHI and RDI scores did not differ between tests, higher RDI scores in both Somte and PSG is consistent with other studies reporting higher RERA's in pregnant women [20]. Given the implications of RERA’s in endothelial dysfunction [21, 22], Somte represents an opportunity to detect increased upper airway burden in pregnant women, in early to mid-gestation, without requiring an overnight stay in the sleep laboratory.

Whilst our study aim was not to assess movement disorders of sleep during pregnancy, one participant in this study was diagnosed with RSWA based on PSG findings. Additionally, this participant demonstrated increased chin EMG during REM in the Somte study, suggesting this incidental finding was replicated in the home environment. The participant reported no history to suggest a presence of a movement disorder and we did not use extended EMG (SINBAR) protocol during the study collection, however the reporting physician determined sufficient evidence on video and polysomnography for a clinical determination of RSWA. The significance of this incidental polysomnographic finding is unclear.

The main limitation of this study was a small sample size. Low study participation rates and high rates of failure to attend after consent, resulted in a lower-than-expected number of participants. This study was completed during a period of political and social instability due to the SARS COV2 pandemic and access to patients was restricted several times through the collection period, so it was unsurprising that we encountered difficulty. In other sleep diagnostic validation studies in pregnant women, conducted in later gestation, similar sample sizes are generally reported. A study investigating home PSG and simultaneous wrist worn Watch-PAT 200 in pregnant women with gestation ≥ 28 weeks, achieved a sample size of 31 [23]. A further study investigating attended PSG and simultaneous ARES Unicorder, in pregnant women 28.6 ± 6.3 weeks gestation achieved a sample size of 16 participants [24]. A study which compared limited channel Apnealink to PSG in late gestation (≥ 28 weeks) within a 14 day window between tests, achieved a sample size of 30 participants [5]. The largest validation study in late gestation is a multicentre study investigating level III Alice PDX compared to attended PSG, in the third trimester, which achieved a sample size of 149 participants, though demonstrated poor agreement in AHI scores [25].

In early to mid-gestation, the largest study investigating agreement with attended PSG, is reported by our group. Our study, which validated Level III Apnealink Air compared to attended PSG in pregnant women around 14 weeks gestation, achieved a sample size of 49 participants and demonstrated good agreement in AHI compared to PSG at AHI ≥ 5 and included participants with severe OSA on PSG (range 0.0–119.7) [4]. Together, our validation studies present two diagnostic test options for use in the diagnosis of OSA in pregnant women in the early to-mid gestation period. We were unable to confirm the validity of the Somte in severe OSA cases in pregnancy and note the overall poorer performance of Somte to detect OSA using RDI. Larger studies to validate Somte in severe OSA, and to verify the performance of the RDI using the Somte device would be beneficial. Due to the study limitations including small sample size, technical issues, and absence of severe OSA amongst study participants, further studies verifying these findings would be beneficial prior to translating these findings into clinical practice.

## Conclusion

Self-applied, unattended Somte was an acceptable substitute to attended PSG in the identification of OSA in pregnant women in early to mid-gestation in this small sample though may fail to detect OSA in some women, particularly when using RDI scores. This provides an opportunity to improve early access to diagnostic testing in the early to mid-pregnancy as recommended for at risk women.

### Supplementary Information

Below is the link to the electronic supplementary material.

Supplementary file1 (DOCX 20 KB)

## Data Availability

Data will be made available on reasonable request.
